# Gold nanoparticle arrays using pre-patterned silicon substrates

**DOI:** 10.1186/s11671-026-04580-z

**Published:** 2026-04-22

**Authors:** Gabriele Schmidl, Marco Diegel, Weixuan Li, Annett Gawlik, Frank Schmidl, Jan Dellith, Uwe Hübner, Jonathan Plentz

**Affiliations:** 1 Leibniz Institute of Photonic Technology (Leibniz-IPHT), Albert-Einstein- Str. 9, 07745 Jena, Germany; 2 Institute of Solid State Physics, Friedrich Schiller University, Helmholtzweg 5, 07743 Jena, Germany

**Keywords:** Thermal dewetting, Gold nanoparticle array, Pre-patterned silicon substrates, Crystallite and particle sizes, Electron microscopy, X-ray diffraction

## Abstract

This work demonstrates a method for producing well-ordered gold nanoparticle arrays using a pre-patterned silicon substrate. The study starts with an investigation of particle formation on flat silicon substrates. Surface coverage is presented as a function of different annealing temperatures (from unannealed to 1100 °C), and particle size distributions are characterized at 1100 °C to study first the annealing process. Silicon substrates pre-patterned with square features were also coated with gold layers ranging in thickness from 10 nm to 40 nm. The dewetting process was then induced by annealing in a furnace at 800 °C and 1100 °C. These varying temperatures and layer thicknesses revealed that a 20 nm initial gold layer deposited onto a square lattice and annealed at 1100 °C resulted in a well-defined nanoparticle array, in contrast to results obtained on flat substrates. Crystallite sizes, determined by X-ray diffraction were smaller than or equal to the smallest determined particle size. Optical absorbance spectroscopy measurements revealed shifts in spectra associated with the plasmonic properties of the nanoparticles.

## Introduction

By controlling not only the shape and size but also the position and arrangement of metal nanoparticles (NP), unique optical, electronic or catalytic properties can be tuned for the application. The arrangement of metal nanoparticles in arrays is a challenging task using it for many applications. For example, they can be used for defined binding detection of chemical or biological molecules to the nanoparticles in localized surface plasmonics [1, 2, 3], for special defined amplification of radiation by the particles [4], as effective catalysts for various chemical reactions or as metamaterials [5, 6]. Differences in NP properties when deposited on glass or silicon wafers can impact the performance and suitability for these applications. Thus, metal nanoparticles typically adhere better to silicon wafers than to glass substrates due to the formation of chemical bonds between the metal and silicon. Furthermore, silicon substrates are ideal for chip-based components in microtechnology due to their ability to be precisely patterned. Since silicon wafers are semiconductors contrary to glass, which is an isolator, the electrical behavior of the metal NP can be affected. There can be a transfer charge between both. The glass is transparent and isolating, thus it is advantageous for plasmonic or in general optical applications. The higher thermal conductivity of silicon than glass can influence the heat transfer between NP and substrate.

Different methods are commonly employed to produce metal nanoparticles [7]. These include for instance chemical synthesis [8] and laser-based ablation in liquid [9, 10]. Both methods are typically followed by either a dripping or spinning process of the NP solution. The arrangement of the NP relies on self-organization on the substrate surface. In contrast, lithographic techniques can produce highly ordered particle structures [11, 12]. However, this method is significantly more expensive and has a low throughput. The starting point for this is a very thin layer of plasmonically active metals such as Au, Ag, Cu or Al with their dielectric and structural properties [13], which are then transformed into plasmonic structures.

Another way for nanoparticle production is the modification of thin films. To bring the energy into the film-substrate system under ambient conditions a laser can be used. A pulsed laser with sufficient energy density, tailored to the absorption characteristics of the thin film material, can elevate temperatures beyond the melting point, approaching the ablation threshold [14]. The breakdown of a metal layer into nanoparticles, known as laser-induced dewetting, occurs at laser fluences exceeding the metal’s melting point. This process is facilitated by the conversion of input energy to heat through collisions between excited electrons, the lattice, and other electrons during the laser pulse [15, 16].

A simpler method is the thermal based solid state dewetting in a furnace [17, 18]. This dewetting process is driven by the reduction of the surface energy of the metal film and the interface energy between layer and substrate surface [19, 20]. This physical phenomenon is based on the rupture of a thin film on a substrate surface up to island and particle formation by increasing the layer system temperature. Below the melting temperature of the metal, the process of solid state dewetting proceeds by surface diffusion. Wang et al. [21] has explored a way to enhance the 2D particle positioning by carrying out the thermal dewetting on pre-patterned silicon wafer substrates with an SiO_2_ layer. He used pyramidal and circular hole structures. Using a SiO_2_ layer has the advantage that caused by a weak interaction between gold and SiO_2_ the atom migration is supported.

Particle formation on a flat substrate typically results in inhomogeneous particle size distributions, with characteristics strongly dependent on the energy input (temperature), annealing time, and initial film thickness [22, 23]. Therefore, the objective of this publication is to demonstrate the feasibility of creating ordered nanoparticle arrays specifically using pre-patterned silicon substrates with square features. Thermal dewetting in a furnace was employed to control particle evolution, with the goal of generating well-aligned gold nanoparticles (Au-NPs). The used square pattern is intended to promote uniform interparticle spacing. Spherical nanoparticle morphology is crucial for achieving narrow absorption bands and, consequently, a narrow resonance frequency within the visible spectral range. Thus, precise control of size distributions and optical properties is essential for applications such as investigations of biological binding events and chemical reactions. Silicon substrates are particularly advantageous for the development of chip-based components.

## Materials and methods

### Substrate patterning

**Fig. 1:**
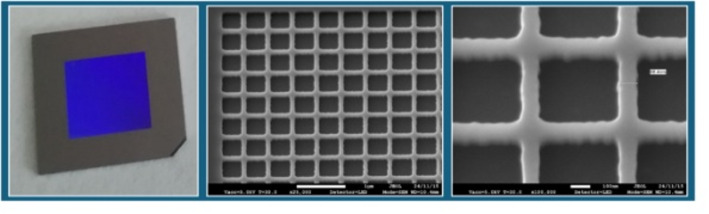
Left: Photo of an 18 × 18 mm chip with a centered 10 × 10 mm patterned area; middle/right: SEM images of the Si etched grid structure in the middle of the chip area. Scale bar: 1 μm and 100 nm

A 4-inch (100) Si wafer was used to fabricate the pre-patterned substrates. The wafer layout contained 16 chips measuring 18 × 18 mm^2^, each with a central 2D grating structure of 10 × 10 mm^2^. The 2D gratings with a pitch of 500 nm and land width of approx. 100 nm were produced by electron beam lithography (Vistec SB350 OS, shaped beam system) in a 200 nm thick ARP6200 resist (Allresist GmbH). These patterns were etched 100 nm deep into the silicon surface using an ICP plasma etching process (device: Sentech SI-500 F, etching gases CHF_3_ and CF_4_). After Si-etching and removal of the remaining resist, the wafers were cut into chips so that the individual experiments could be carried out. Figure 1 shows an example of the prepared chip and structure.

### Preparation of gold films

The pre-patterned substrates were cleaned and featured a thin native silicon oxide layer of approximately 5 nm thickness before gold deposition. To ensure consistent ambient environmental conditions across all experiments, the conventional HF dip removal process was intentionally avoided, preserving the native oxide layer [24].

**Fig. 2:**
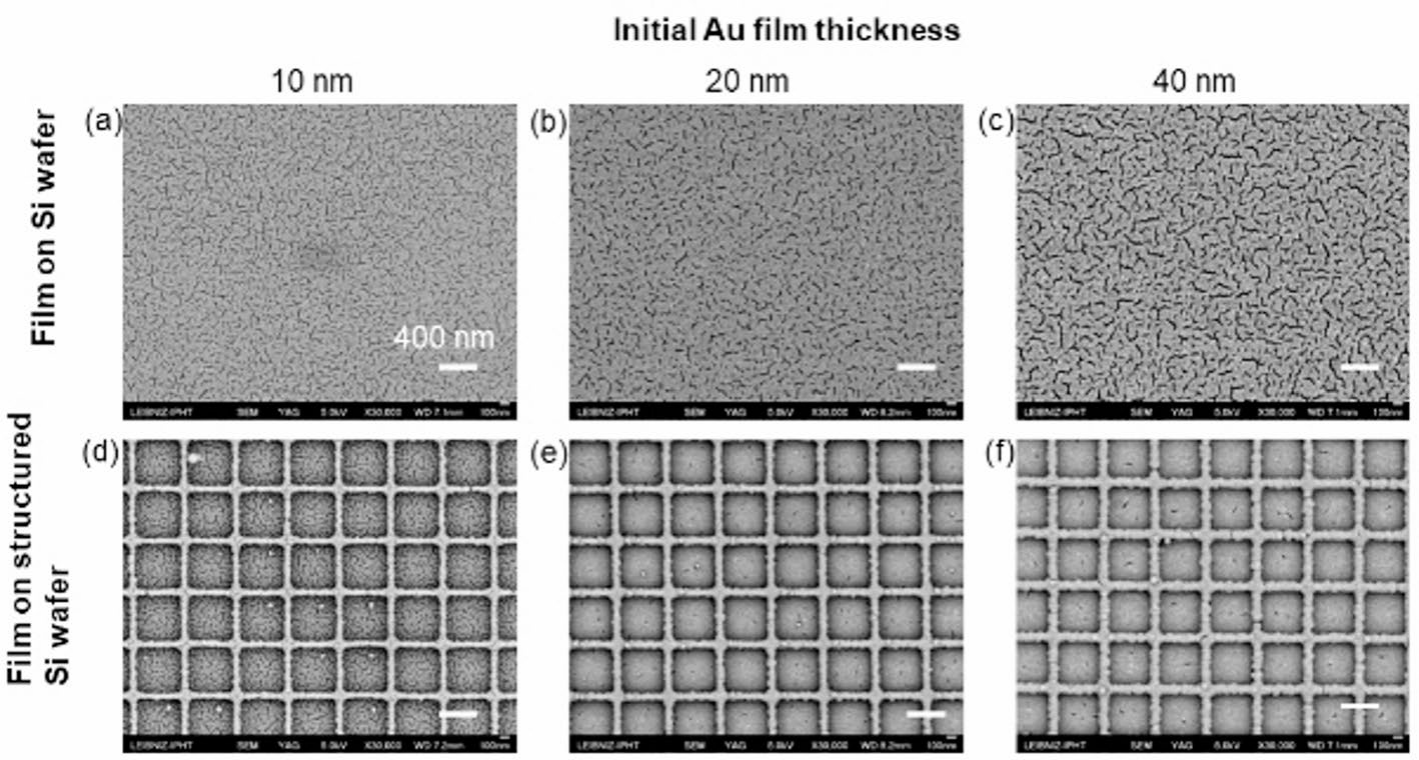
SEM images of the coated substrates; *top*: Si wafer substrate without structure, **a** 10 nm, **b** 20 nm, **c** 40 nm Au film; *bottom*: patterned and coated Si wafer, **d** 10 nm, **e** 20 nm, **f** 40 nm Au film, scale bars: 400 nm

For the experiments, gold layers with thicknesses (h_Au_) ranging from 10 to 40 nm were deposited onto these substrates using a sputter coater. The deposition process was conducted at a working pressure of 2 × 10^−1^ mbar, with a voltage of 1.3 kV, a current of 15 mA, and a deposition rate of 0.24 nm/s. In Fig. 2 the coated Si wafer substrates with and without the patterned area are presented. The SEM images in the backscattering (BSE) mode, taken with a YAG (Yttrium aluminum garnet) detector providing high-resolution and good signal-to-noise ratio, show the differently pronounced surface structures dependent on the film thickness and the more or less occupied area within the squares. In the 10 nm layer, the film on the ridges is also not closed - in contrast to the 40 nm layer.

### Thermal annealing via furnace

The thermal annealing process was performed using a Heraeus tube furnace. Substrates were positioned on a ceramic holder in the center of the furnace to have homogeneous temperatures. The samples were heated in air for 60 min at 200 °C, 500 °C, 800 °C and 1100 °C and the pre-patterned samples were heated at selected temperatures of 800 °C and 1100 °C. The chosen annealing time of 60 min corresponding to the used temperatures is sufficient to enable surface tension-driven reorganization of the thin film.

### Surface investigations

The formed Au structures on the surfaces were visualized using scanning electron microscopy (SEM). An FEI Helios NanoLab G3 UC (ThermoFisher Scientific) and the JSM-6700 F from JEOL Ltd were used for this. The resulting images were the basis for the determination of NP size distributions with the open source image analysis software ImageJ (Java-based image processing program–LOCI, University of Wisconsin) combined with ORIGIN (OriginLab Corporation).

The crystallographic structure of the gold thin films was investigated using X-ray diffraction (XRD). Measurements were performed with a diffractometer Panalytical Xpert Pro equipped with a long-fine-focus copper X-ray tube (Cu Kα radiation, λ = 1.5406 Å). To ensure high-resolution data acquisition and minimize beam divergence, the incident beam optics included a parallel-beam mirror, a ½° divergence slit, a 10 mm beam mask, a 0.04 rad Soller slit, and a 1.4 mm anti-scatter slit. The diffracted beam optics were a 7.5 mm fixed anti-scatter slit, a large 0.04 rad Soller slit and a PIXCEL3D Silicon area detector in 1D mode with 255 channels. This configuration allowed for precise control of the beam geometry and reduction of background noise, thereby enhancing the signal-to-noise ratio in the diffraction patterns. The samples were horizontally positioned, and scans were conducted in θ–2θ geometry. Data were collected over a suitable angular range to capture the characteristic diffraction peaks of the gold films. An offset angle of 2° was used to suppress the silicon peaks by some order of magnitudes. The resulting diffraction profiles were analyzed to determine the crystallite size by a linear Williamson-Hall plot (W-H plot) analysis. Quantitative analysis of surface coverage and particle size distribution from SEM images was performed using ImageJ (Java-based image processing program–LOCI, University of Wisconsin), an open-source image processing software. The SEM micrographs were imported into ImageJ and converted to 8-bit grayscale format to facilitate image segmentation. A suitable manual thresholding was applied to distinguish particles from the background. To enhance segmentation accuracy, preprocessing steps such as contrast adjustment or noise reduction were employed where necessary. For surface coverage analysis, the total area of the segmented material area was measured and normalized to the total image area. Particle size distribution was determined by quantifying the area of individual particles and the diameter determination assuming spheres. Particle counting and the mean particle size were analyzed using Origin2024b (OriginLab Corporation).

### Optical investigations

The optical measurements were carried out in reflection. Illumination was provided by a fiber light source (Avalight-DH-SAvantes), which generated an elliptical spot under 45° on the sample of approx. 2 × 3 mm. A USB-fiber-spectrometer (Ocean Optics) was placed at an angle of 90° to detect the reflected light. The sample was precisely positioned using an x-y translation stage.

## Results and discussion

### Nanoparticle formation on a flat silicon substrate

**Fig. 3:**
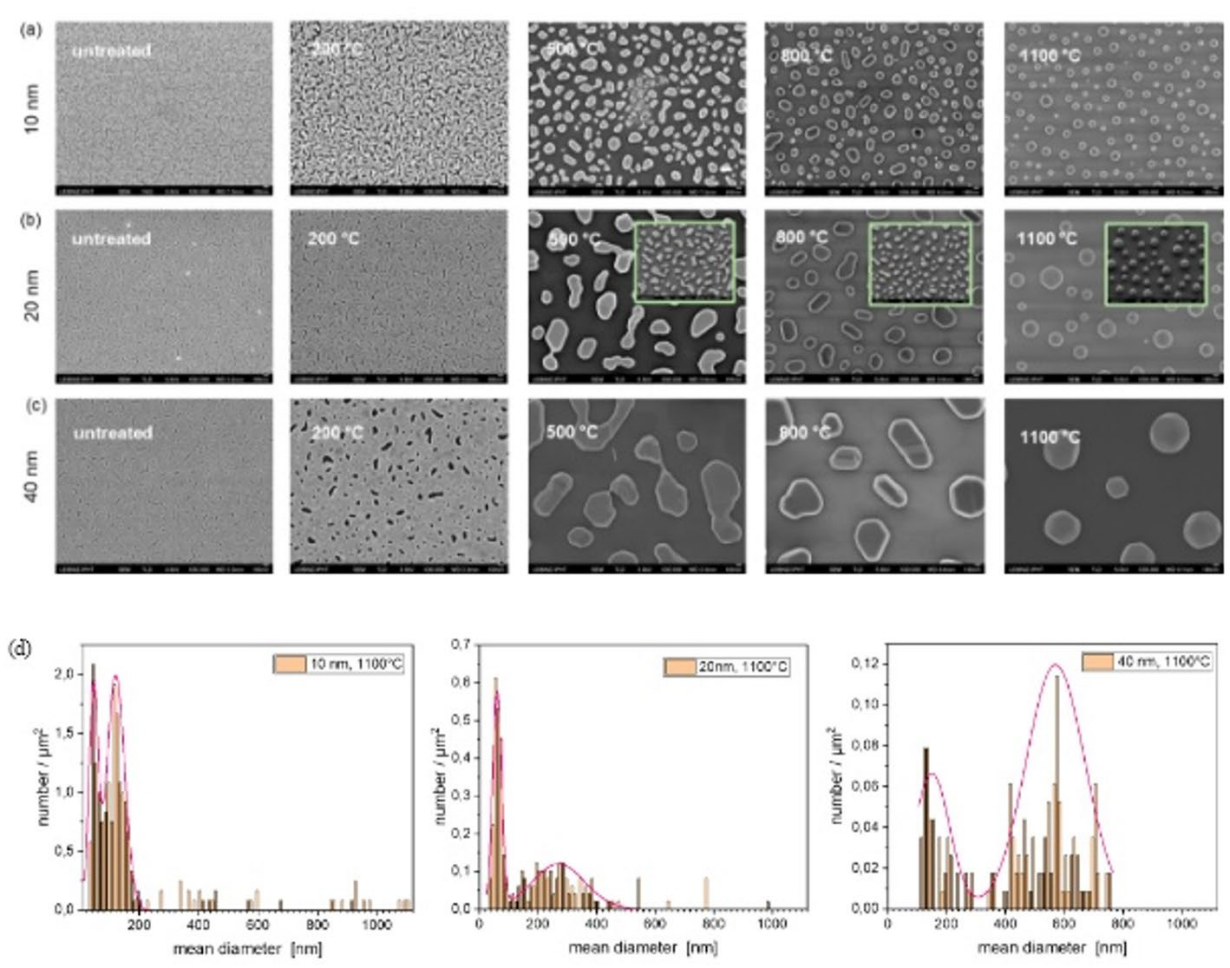
SEM images (TLD) of untreated and annealed (200 °C, 500 °C, 800 °C, 1100 °C) Au films on flat Si substrates; **a** 10 nm, **b** 20 nm, insets: tilted SE images showing the 3D evolution, **c** 40 nm Au film thickness, magnification: x30000; scale bars: 100 nm; **d** Particle size distributions for 10, 20 and 40 nm Au layer with annealing at 1100 °C

When flat Si substrates coated with a thin metal layer are exposed to a certain temperature, the surfaces become de-networked, leading to the formation of islands or particles. The result of this process, i.e., the shape and size of the islands or particles, depends on the ambient temperature, the layer thickness, and the time. Gold is an inert metal that does not react with air but is affine to silicon. This multifaceted dependency is well described in the literature [18, 25]. In this publication, we have focused on the layer thickness and temperature dependency. On a flat substrate, this dewetting process develops as shown in Fig. 3a–c. Starting from a deposited layer that is permeated with holes and grooves, the layer becomes coarser as the temperature rises, which is associated with larger holes, until individual islands are formed. These islands are rather irregularly shaped 2D structures. The surface coverage determined by the image analysis software is reduced from 0.78 to 0.91 per µm^2^ to ~ 0.15 per µm^2^ (Table 1).

That could indicate the evolution of single particles that are more three dimensional (see insets Fig. 3b, tilted SE images). As seen in Fig. 3a–c for 1100 °C the resulting structures can be described as regular 3D spheres at a temperature of 1100 °C. Therefore, the particle size distributions dependent on the initial layer thickness was determined (Fig. 3d). It shows, first, a bimodal distribution and, second, bigger particles per µm^2^ with increasing layer thickness. Based on this observation of particle formation on a flat substrate, it can be assumed that particle formation with a 3D character occurs at temperatures close to the melting point of Au. Therefore, only temperatures of 800 °C and 1100 °C were used for investigation and analysis of particle formation on the pre-patterned substrates.

**Table 1:** Surface coverage for different initial layer thicknesses dependent on annealing temperature normalized to µm^2^, particle size distribution for 10, 20 and 40 nm Au layer after annealing only for 1100 °C assuming a sphere (see Fig. 3)

T [°C]	d =10 nm		d=20 nm		d = 40 nm	
	Covered area per µm²	Mean particle diameter, peak position (1/2) [nm]	Covered area per µm²	Mean particle diameter, peak position (1/2) [nm]	Covered area per µm^2^	Mean particle diameter, peak position (1/2) [nm]
non	0.78	–	0.88	–	0.91	–
200	0.66	–	0.86	–	0.87	–
500	0.28	–	0.26	–	0.27	–
800	0.17	–	0.23	–	0.24	–
1100	0.14	50/120	0.13	50/300	0.16	120/580

### Nanoparticle formation on pre-patterned substrates

**Fig. 4:**
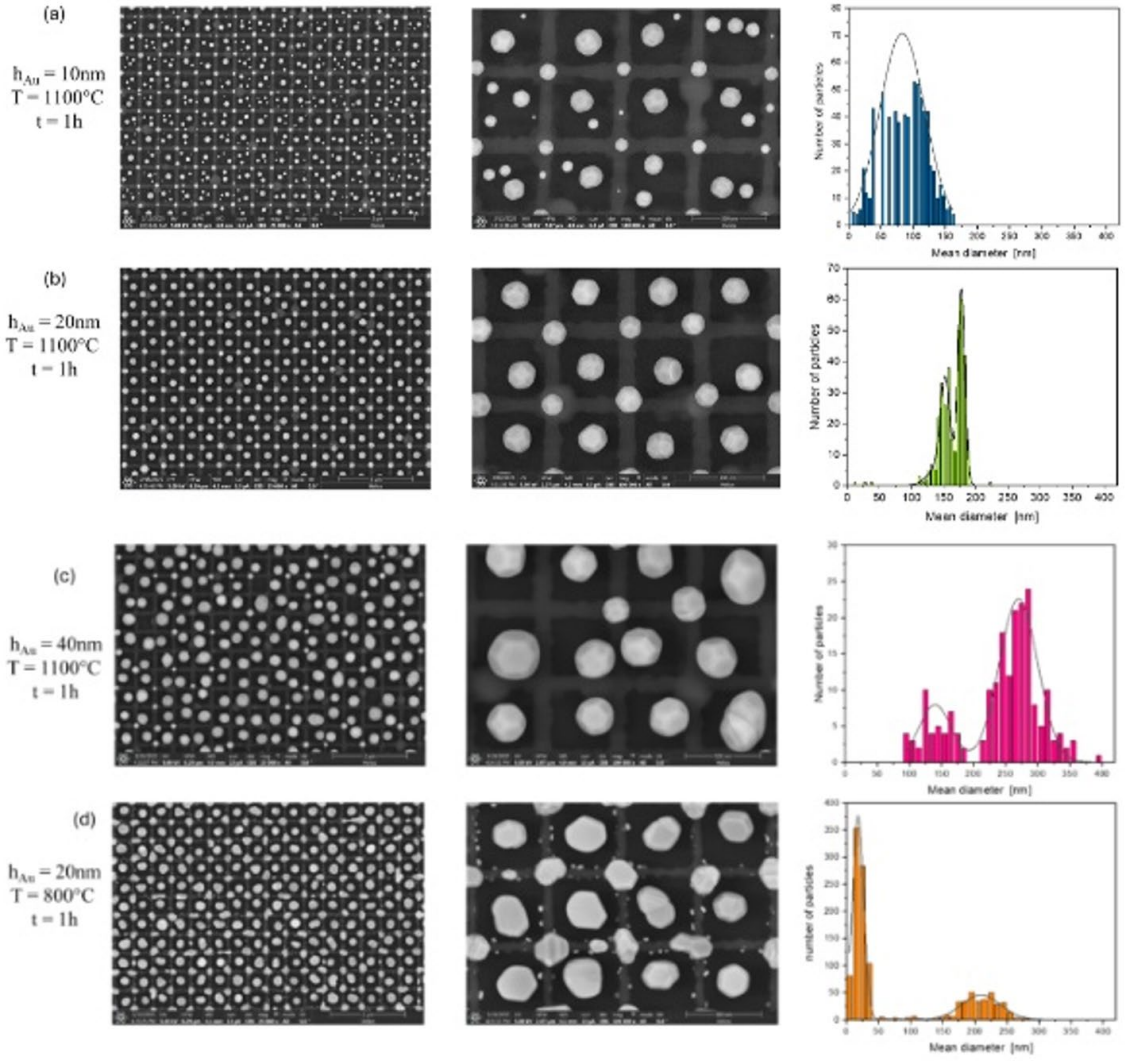
Particle formation after thermal treatment in a furnace of different thick original Au layer, **a** 10 nm, **b** 20 nm, **c** 40 nm) @1100°C and **d** 20 nm @800°C. The scale bar is 2 μm or 500 nm respectively

It is possible to control particle formation by specifically influencing the dewetting kinetics through pre-defined structures and thus by locally influencing surface energies [26]. The pre-patterned topography is utilized to guide the formation of particles. In addition to the structure, the used film thickness of the dewetting material in our case Au and the annealing temperature are also important for array formation. Therefore, the objective in this publication was to generate an array of gold nanoparticles and to observe how they are influenced by the furnace temperature and the thickness of the gold layer. In Fig. 4 it is clearly observed that the Au particle formation is guided by the silicon structure. Particles exist preferred on the crossing points of the silicon grid and in the squared grid area. Since the solid-state dewetting is initiated either at a pre-existing edge of the film for instance caused by an edge of the Si structure or through formation of holes that create edges, an increase in Au film thickness using the highest annealing temperature of T = 1100 °C, which means more film volume for dewetting, leads to different dewetting results. For h_Au_ = 10 nm Au film thickness, small particles are formed on each grid crosspoint (Fig. 4a). These particles are smaller one and show a broad size distribution with a mean diameter d in the range of 40–60 nm. On the squared regions, the film becomes dewetted to form structures consisting of a few particles of different sizes and spacings (Fig. 4a). The sizes can be found in the range of 100–120 nm or bigger and the smaller one at around 20 nm. However, these distributions merge almost seamlessly into a broad distribution with a mean dimeter at d_mean1_ = 80 nm. The calculated size distribution is presented in Fig. 4a on the right side (blue bars). The higher Au film thickness of h_Au_ = 20 nm leads to absolutely ordered arrays of particles with particles of near the same size (Fig. 4b). Right to the SEM images a diagram of the particle size distribution from the left SEM image in Fig. 4b is shown. Here, one can observe significantly a bimodal but small distribution with maximums at d_mean1_ = 150 nm and d_mean2_ = 180 nm, or, when considering some smaller particles, even a trimodal distribution (additionally a maximum at d = 130 nm). In this constellation, the particles with the larger mean diameters are arranged on the squared surface, whereas the somewhat smaller ones are found at the intersections of the squared structure. If the thickness of the original Au film is increased further to h_Au_ = 40 nm, an increase in the mean diameter and a broadening of the bimodal bands at d_mean2_ = 270 nm and d_mean1_ = 130 nm is recognizable (Fig. 4c). This behavior is observed using the highest annealing temperature of 1100 °C that is around the melting temperature of the gold. If the particle formation at 1100° is compared with that at 800 °C, i.e. well below the melting point of gold, not only the particles become more irregular, but the average particle diameter is also larger (d_mean2_ = 210 nm). In addition, much smaller residual particles with a size of d_mean1_ = 20 nm remain, which are mostly found at the structure edges.

All the regular particles formed at 1100 °C during 1 h show faceting. At 800 °C this can only be seen to some extent (Fig. 4d). In contrast to Fig. 4b, it is clearly evident that faceting of the larger particles has not yet occurred, nor has a tendency towards regular arrangement, although this is not yet fully developed. The particle size distribution reveals the presence of both very small particles with an average diameter of 20 nm and significantly larger particles with an average diameter of 210 nm. These larger particles do not yet exhibit a perfect spherical morphology. The presence of two distinct particle size populations suggests a complex growth or aggregation process. The fact that the larger particles are not perfectly spherical indicates they can be potentially in an intermediate stage of development.

Furthermore, it should be noted that the reproducibility of the array formation is strongly influenced by the structural fidelity and uniformity of the substrate. Once the substrate patterns are fabricated with high precision, the resulting particle arrays are consistently reproduced under comparable experimental conditions, even though individual particle positions may vary slightly and faceting may show minor differences; the size distribution, however, remains very good within this range.

The time dependence of the dewetting process on a pre-patterned substrate was not investigated here. However, it can be assumed that the dewetting result and thus the particle diameter and shape also depend on this parameter on such a substrate. In addition to the surface properties of the substrate, temperature and time are the interdependent influencing variables for the result of dewetting. The modeling of dewetting and coarsening phenomena is a subject of ongoing research. For instance, [27, 28] presented a ripening model for the self-organization of nanoclusters, building upon the coarsening theories of Baldan, Lifshitz-Slyozov, and Allmang-Feldman [29–31]. These models demonstrate that the resultant nanoparticle radius (r) (Eq. 1) is dependent on time (t), temperature (T), initial nucleation density (N₀), atomic diffusion coefficient (D), surface energy (γ), film thickness (L), and atomic volume (Ω) [32].

1 $$r\left( {T,t} \right)^{n} \sim ~K\left( T \right) \cdot t\;{\mathrm{with}}\;K\left( T \right)\sim N_{0} \cdot D\left( T \right) \cdot \frac{{{{\Omega }}^{2} }}{T} \cdot \ln \left( L \right) $$

However, this equation may not be directly applicable here. On patterned substrates, the dewetting dynamics are governed by additional factors including topographic confinement, local variations in film thickness due to substrate curvature, and anisotropic surface diffusion along patterned features. These effects would require substantial modification of the theoretical framework and could make the theoretical relationship more complex.

### Material and structure investigations of nanoparticles on patterned substrates

**Fig. 5:**
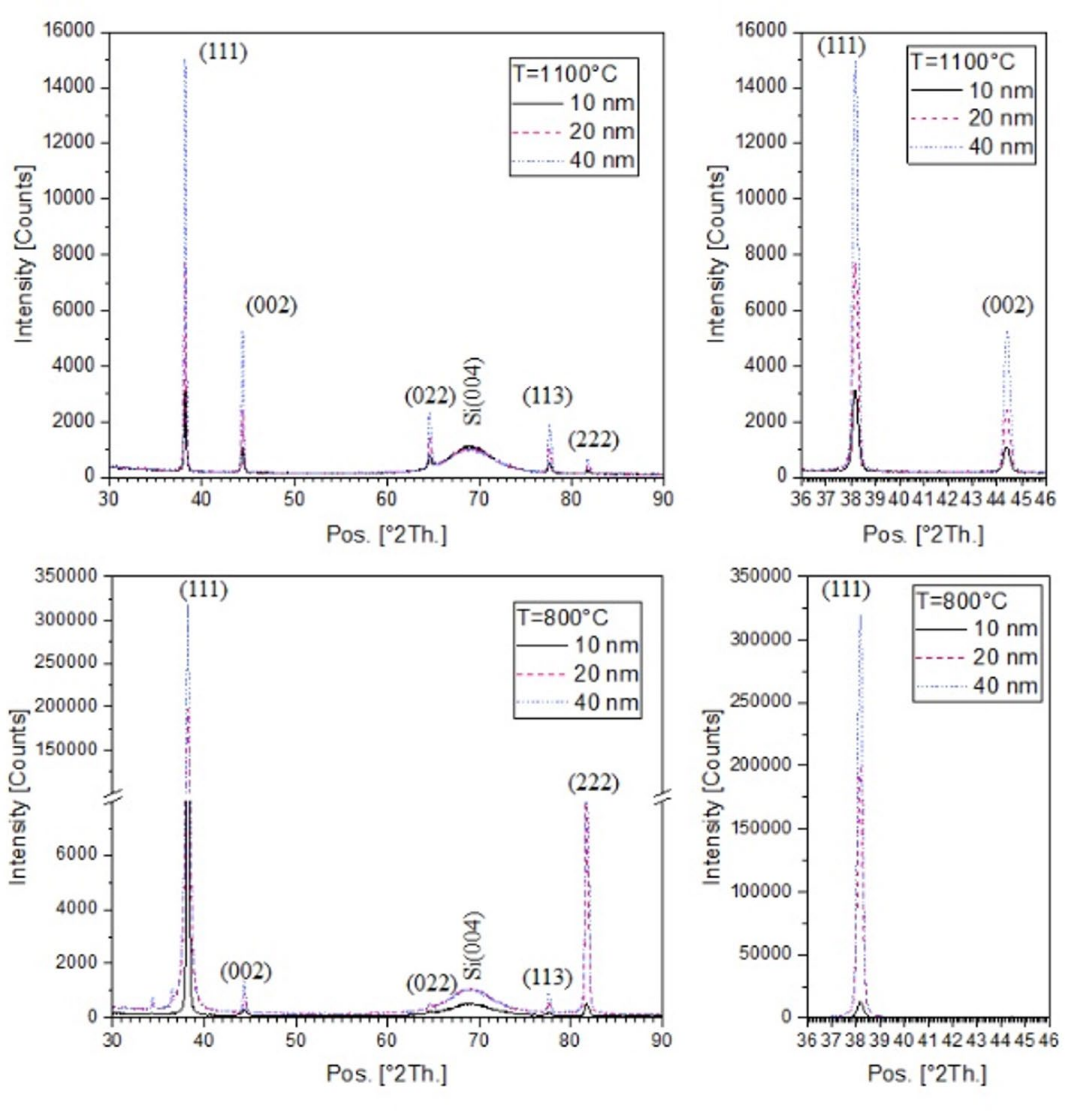
X-ray diffraction spectra with (111), (002), (022) and (113) reflexes at 38.2°, 44.4°, 64.6°, 77.6°, 10 nm, 20 nm and 40 nm initial Au layer thickness annealed at 800 °C and 1100 °C, the broad (004) reflex of the silicon (001)- substrate was suppressed, due to 2° offset in the 2Theta-omega-scan

To investigate facet development and to investigate the crystallite size of the particles, X-ray diffraction and Williamson-Hall analysis were performed. Samples annealed at 1100 °C exhibited a polycrystalline nature, confirmed by the presence of multiple orientations ((111), (002), (022), or (113) reflexes at 38.2°, 44.4°, 64.6°, 77.6°). Varying reflex intensities suggest differing initial layer thicknesses (Fig. 5). Crystallite sizes, determined from the spectra, correspond to the range of smallest average particle diameters. Consequently, larger particles with greater average diameters contain a higher number of grains with different orientation (see Table 2).

Below gold’s melting temperature (1064 °C), as is the case here at 800 °C applying 1 h, facet development is not yet pronounced, and the particles tend towards a planar morphology. The spectra reveal a significant (111) preferred orientation but a small (002) reflex in this case, contrary to 1100 °C annealing. The intensity ratio of (111) to (002) reflex for all three different layer thicknesses is 2.9 ± 0.2 using 1100 °C and 223 ± 9 for 800 °C. The absolute reflex intensity in the 2Theta scan is higher in case of 800 °C annealing. This orientation difference indicates that the nanoparticles are growing in a way that minimizes surface energy, leading to specific shapes, anisotropic properties. The kinetic energy isn’t high enough for 800 °C to overcome energy minimization and allow for the development of other facets, hence the pronounced (111) orientation.

**Table 2:** Crystallite size determined with W-H plot using spectra of Fig. 5 and mean peak position of the size distribution with respect to Fig. 4 only for 1100 °C when spheres can be assumed

h_Au_ [nm]	T [°C]	Crystallite size	Mean diameter/peak position (patterned substrat)	
		[nm]	d_mean1_ [nm]	d_mean2_ [nm]
10	1100	56 ± 12	80	–
20	1100	133 ± 23	150	180
40	1100	137 ± 30	130	270
10	800	28 ± 7	–	–
20	800	64 ± 19	–	–
40	800	70 ± 22	–	–

Especially the (111) orientation, frequently observed and often dominant in specific growth conditions of metal thin films and can introduce anisotropy to the nanoparticle’s shape as facets (compare Fig. 4). The (100) facets are more likely to appear when growth conditions favor kinetic control over thermodynamic equilibrium, i.e., at higher temperatures or faster growth rates where atoms do not have enough time to arrange themselves into the lowest-energy configuration. That is seen comparing the X-ray spectra for 1100 °C and 800 °C annealing temperature (Fig. 5).

### Optical characterization of gold nanoparticles on flat and pre-patterned substrates

**Fig. 6:**
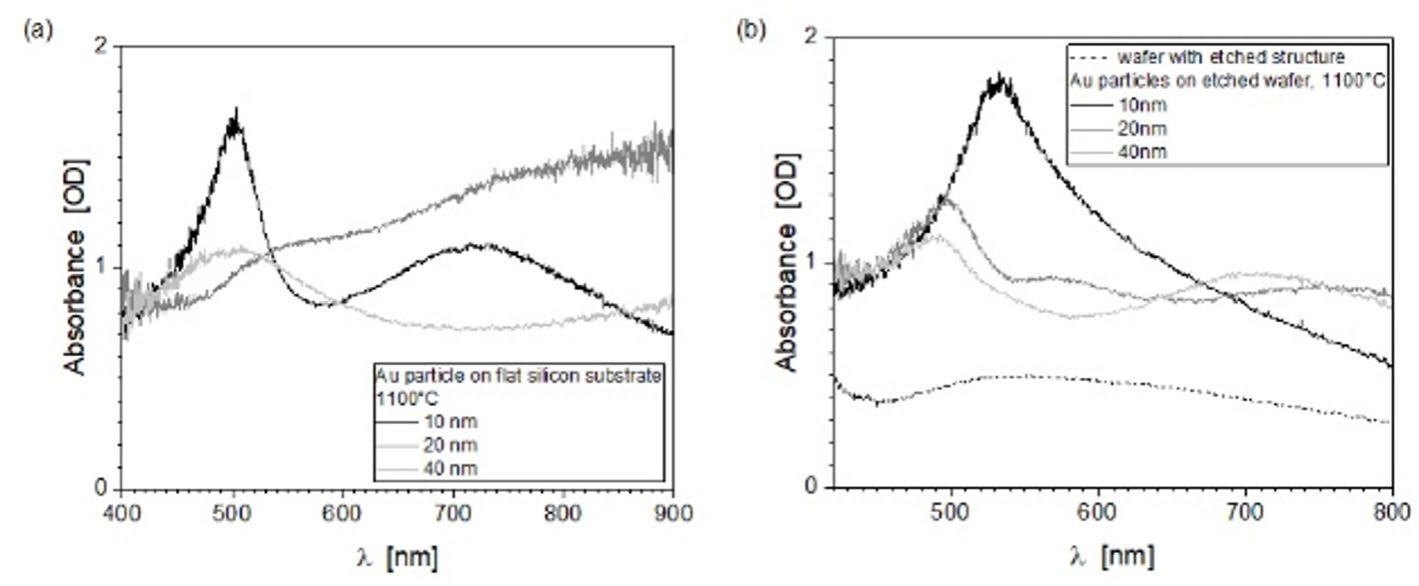
**a**,** b** Optical response of particles formed on flat substrates in comparison to the silicon wafer with etched structures, Spectra using 10, 20 and 40 nm Au films and only the pre-patterned substrate (black curve)

Since almost round, faceted particles are formed at high annealing temperatures, the processes with layer thicknesses of h_Au_ = 10, 20 and 40 nm and a temperature of 1100 °C will be considered optically further for the furnace process. When evaluating absorbance, the opacity of silicon within the visible spectral range must be considered. Consequently, the spectral response was initially characterized via reflection measurements performed on particles deposited on both flat and pre-patterned substrates, and normalized to a flat silicon substrate. Figure 6 presents a comparison of these curves, demonstrating the influence of the substrate structure and the different particle formation results including different size distributions. When identifying resonance positions and bandwidth, it is important to consider the size and distribution of particles, as well as the characteristics of facets forming at 1100 °C annealing temperature. These facets can influence resonance formation in addition to the other mentioned properties due to different plasmon modes along the anisotropic axes. Thus, the facet formation can introduce additional complexity to the LSPR spectra, potentially broadening peaks and reducing polarization.

The LSPR spectra of the particles on flat wafers (Fig. 6a), regardless of layer thickness (10, 20 and 40 nm, 1100 °C), show several resonance modes, with the modes being most pronounced and closest together for the 10 nm Au layer. This indicates very small particles with a narrow particle distribution and, in addition, larger particles with a broader particle size distribution. For 20 and 40 nm Au layer thicknesses, there are broader size distributions and significantly larger particles, as presented in the SEM images in Fig. 3.

The particle-loaded structures were measured against the flat wafer, so spectra still contain the structural influence. The absorbance spectrum of the pre-patterned substrate without particles (Fig. 6b, *black curve*) exhibits a maximum at around 540 nm and a small broad spectral response contrary to a flat Si surface [33]. For the other spectra (Fig. 6b), including those with Au nanoparticles on the substrates, the corresponding SEM images can be found in Fig. 4.

Particles forming from a 10 nm Au layer show a resonance maximum at 540 nm. This can be assigned to the mean diameter of the particles produced of d_mean1_ = 80 nm (Table 2). Nevertheless, the spectrum is broadened by particle size distribution. For h_Au_ = 20 nm and 40 nm a multimode curve was found, and an amplification effect and a resonance shift due to bigger particles can be recognized corresponding to the size distributions.

Especially (111) facets contribute to these distinct resonances. But the (100) orientation, which has a higher surface energy than (111), but is less favored during nanoparticle growth as the system tends to minimize energy. Thus, the influence on the LSPR based on (100) facets is more likely to appear at higher temperatures (1100 °C) also seen in the X-ray spectra.

In general, when nanoparticles are arranged into an array, their plasmonic response is determined by several factors, including particle size, size distribution, and the complex electromagnetic coupling between the individual scattering centers. These interactions produce multiple resonant modes that coexist and partially overlap, originating both from the size‑dependent localized surface plasmon resonances of the individual particles and from collective coupling phenomena. Their interplay yields spectral broadening, multimodal resonance signatures, and enhanced near‑field inhomogeneity throughout the array. Additional to spherical particles which have a single, broad LSPR peak, anisotropic particles like faceted particles exhibit multiple LSPR peaks, polarized along different directions. This effect, that leads for instance to a peak split or a wavelength shift, can be seen more clearly when single-particle LSPR investigations are carried out, which was not performed as part of this work. Furthermore, sharp edges intensify local electromagnetic field confinement. These geometry-induced effects have been documented for faceted Au nanorods and related anisotropic structures. In SERS, these facets create intense hot spots at edges and corners, producing significantly stronger electromagnetic enhancement together with facet‑specific adsorption behavior, as shown for faceted Au nanoparticles used in NIR‑SERS substrates [34, 35] An optimal anisotropic design therefore incorporates well-defined facets, sharp edges, high-index planes, and tailored facet orientations.

## Conclusion

This study examines nanoparticle formation on a square pre-patterned substrate, using a planar substrate as a reference. The goal was to generate an ordered array of similarly sized gold particles via the pre-patterning process. It should be noted that the reproducibility of the array formation is strongly influenced by the structural fidelity and uniformity of the substrate structure. Particle formation on a planar substrate is temperature-dependent, with pronounced 3D particle development only occurring at temperatures approaching the melting point. This process was investigated using a fixed annealing time of 1 h. Therefore, the influence of annealing time was not considered in the present study. However, it should be noted that the annealing duration can affect the morphology and size evolution of the resulting nanoparticles. This 3D growth is characterized by facet formation seen in the SEM images. At lower temperatures, planar coarsened islands are observed, associated with a dominant (111) crystal orientation as expected for deposited thin gold films and related to minimizing surface energy. A benefit of facet formation is that the (111) facets can act as “hot spots” with particularly strong electric field enhancement during plasmon excitation. This enhancement can be leveraged for applications such as Surface-Enhanced Raman Scattering (SERS). In addition to facet formation, pre-patterning silicon substrates with a 20 nm gold layer at a temperature of 1100 °C results in an array of gold nanoparticles. The particle size distribution is narrow and bimodal, with the smallest average diameter corresponding to the crystallite size as determined by X-ray diffraction and Williamson-Hall analysis. The larger particles, with an average diameter of 200–300 nm, are assumed to consist of at least two to three crystallites in minimum. In case of a pre-patterning at annealing temperatures near the melting point of gold, the (100) orientation becomes more prominent. The (111) to (002) reflex intensity ratio is significantly lower at 1100 °C (2.9 ± 0.2) compared to 800 °C (223 ± 9). Beside a controlled particle size and size distribution also by controlling the degree of (111) facet development—for example, by adjusting the temperature or growth conditions—the Localized Surface Plasmon Resonance (LSPR) peak position and polarization characteristics of the nanoparticles can be tuned. For optical applications, however, the choice of substrate becomes particularly important. Substrate selection is a critical factor for particle formation, as surface energies strongly influence the dewetting behavior of thin gold films. Gold, for example, adheres less strongly to glass than to silicon, which can lead to modified dewetting dynamics. The formation of well‑faceted, nearly round particles additionally requires annealing temperatures that exceed the thermal stability limits of common glass substrates, whereas alternative transparent materials such as quartz or SrTiO_3_ can withstand these conditions. Another important requirement is that the substrate must allow reliable surface patterning or provide a defined topography, which is not feasible for all materials. Since optical applications generally demand optically transparent substrates, it may therefore be necessary to transfer the particle arrays onto suitable transparent materials or to measure in reflection. The unique capability of our method enables the fabrication of highly ordered periodic structures that are challenging to achieve via conventional techniques yet are crucial for significant advances in plasmonics research and sensor device development.

## Data Availability

All data supporting the findings of this study are available from the corresponding author on reasonable request. Data are located in controlled access data storage at the Leibniz IPHT.
